# Seroprevalence of Dengue Virus and Rickettsial Infections in Cambodian Children

**DOI:** 10.4269/ajtmh.18-0865

**Published:** 2019-01-21

**Authors:** Andrew Fox-Lewis, Jill Hopkins, Poda Sar, Sena Sao, Ngoun Pheaktra, Nicholas P. J. Day, Stuart D. Blacksell, Paul Turner

**Affiliations:** 1 Cambodia-Oxford Medical Research Unit, Angkor Hospital for Children, Siem Reap, Cambodia;; 2 Nuffield Department of Medicine, Centre for Tropical Medicine and Global Health, University of Oxford, Oxford, United Kingdom;; 3 Angkor Hospital for Children, Siem Reap, Cambodia;; 4 Mahidol-Oxford Tropical Medicine Research Unit, Faculty of Tropical Medicine, Mahidol University, Bangkok, Thailand

## Abstract

Scrub typhus (ST, *Orientia tsutsugamushi*), murine typhus (MT, *Rickettsia typhi*), and dengue virus (DENV) are important causes of childhood morbidity in Cambodia. This prospective, cross-sectional seroprevalence study determined the proportion of Cambodian children infected by these pathogens and the ages at which initial infection is likely to occur. A total of 993 patient serum samples were tested for MT- and ST-specific IgG, and 837 samples tested for DENV-specific IgG. Overall, ST, MT, and DENV seroprevalence was high, estimated at 4.2%, 5.3%, and 50.7%, respectively. Scrub typhus and MT seropositivity peaked in children aged 8–11 and 12–15 years, respectively, suggesting initial infection occurs in these ages. Dengue virus seroprevalence steadily increased with age, indicating constant DENV exposure. The results of this study suggest that in Cambodian children presenting with undifferentiated febrile illness, dengue should be considered high in the list of differential diagnoses, and empirical anti-rickettsial antimicrobial therapy may be more indicated in 8- to 15-year-olds.

Scrub typhus (ST, *Orientia tsutsugamushi*), murine typhus (MT, *Rickettsia typhi*), and dengue virus (DENV) are emerging as the major causes of non-malarial febrile illness in Southeast Asia.^1,2^ In a previous study of hospitalized Cambodian children, they accounted for 16.2%, 7.8%, and 2.2% of admissions with febrile illness, respectively.^3^ As non-culturable causes of undifferentiated fever, early diagnosis is challenging, and thus it is vital to understand in which patient subgroups rickettsia and dengue infection occur more commonly, especially because rickettsial infections are easily treatable.^4^ Despite the importance of these pathogens as causes of febrile illness in Cambodian children, it is not known what proportion of children have previously been infected and at which ages initial infection occurs. This study aimed to address these unknowns.

A prospective, cross-sectional seroprevalence study was conducted at Angkor Hospital for Children (AHC), a nongovernmental pediatric hospital in Siem Reap Province, Cambodia. Unselected consecutive leftover serum samples were obtained over 4 months (July 23–November 22, 2017) from the AHC biochemistry-hematology laboratory, one sample per patient. Samples originated from any hospital department, were ordered by the primary clinician for other reasons, and were retrieved after 24–48 hours refrigerated storage before being discarded. Target sample numbers were 60–70 per year of life (birth–15 years). Following retrieval, samples were anonymized and stored at −80°C before study-specific testing. An Excel database (Microsoft Corp, Redmond, WA) was created to record patient age at sample collection, gender, and serology results.

Scrub typhus and DENV IgG were detected using commercially available ELISAs: ST Detect^™^ IgG ELISA System and DENV Detect^™^ IgG ELISA (InBios International, Inc., Seattle, WA). These assays were selected because of their previous evaluation in seroprevalence studies of these pathogens.^5–8^

Murine typhus IgG was detected using an in-house ELISA (Mahidol-Oxford Tropical Medicine Research Unit, Bangkok, Thailand). For each 96-well microtiter plate, *R. typhi* sonicated antigen (whole cell lysate) diluted 1:4,000 in phosphate-buffered saline (PBS) was added to 48 wells, and PBS only added to 48 wells. Plates were stored for 36–48 hours at 4°C, then aspirated and blocked with blocking buffer (5% skimmed milk in wash buffer of 0.1% Tween-20 in PBS) for 1 hour, and washed, before addition of patient sera diluted 1:100 in blocking buffer. After 1 hour room temperature incubation, plates were washed and horseradish peroxidase-conjugated goat-anti-human IgG diluted 1:1,000 in blocking buffer was added. Following 1 hour further room temperature incubation, plates were washed and tetramethylbenzidine substrate added. After 15 minutes room temperature incubation in darkness, stop solution (1 mol/L hydrochloric acid) was added and plates read at 450 nm, minus a reference value at 600 nm. The final optical density (OD) for antigen containing wells was calculated by subtracting the OD from corresponding wells containing no antigen (background absorbance). Phosphate-buffered saline, Tween-20, anti-human IgG, and tetramethylbenzidine were obtained from Thermo Fisher Scientific (Waltham, MA).

Each sample was tested once per assay. For ST and MT, OD frequency distributions were plotted (Figures 1 and 2), and for both assays, the mean OD plus one SD was selected as the cutoff for determining seropositivity. For DENV, a positive result was defined as per manufacturer instructions. Briefly, for each sample, the OD of the ELISA against DENV-recombinant antigen was divided by the OD of the ELISA against normal cell antigen, to generate an immune status ratio (ISR), with an ISR ≥ 2.84 considered positive.

**Figure 1.**
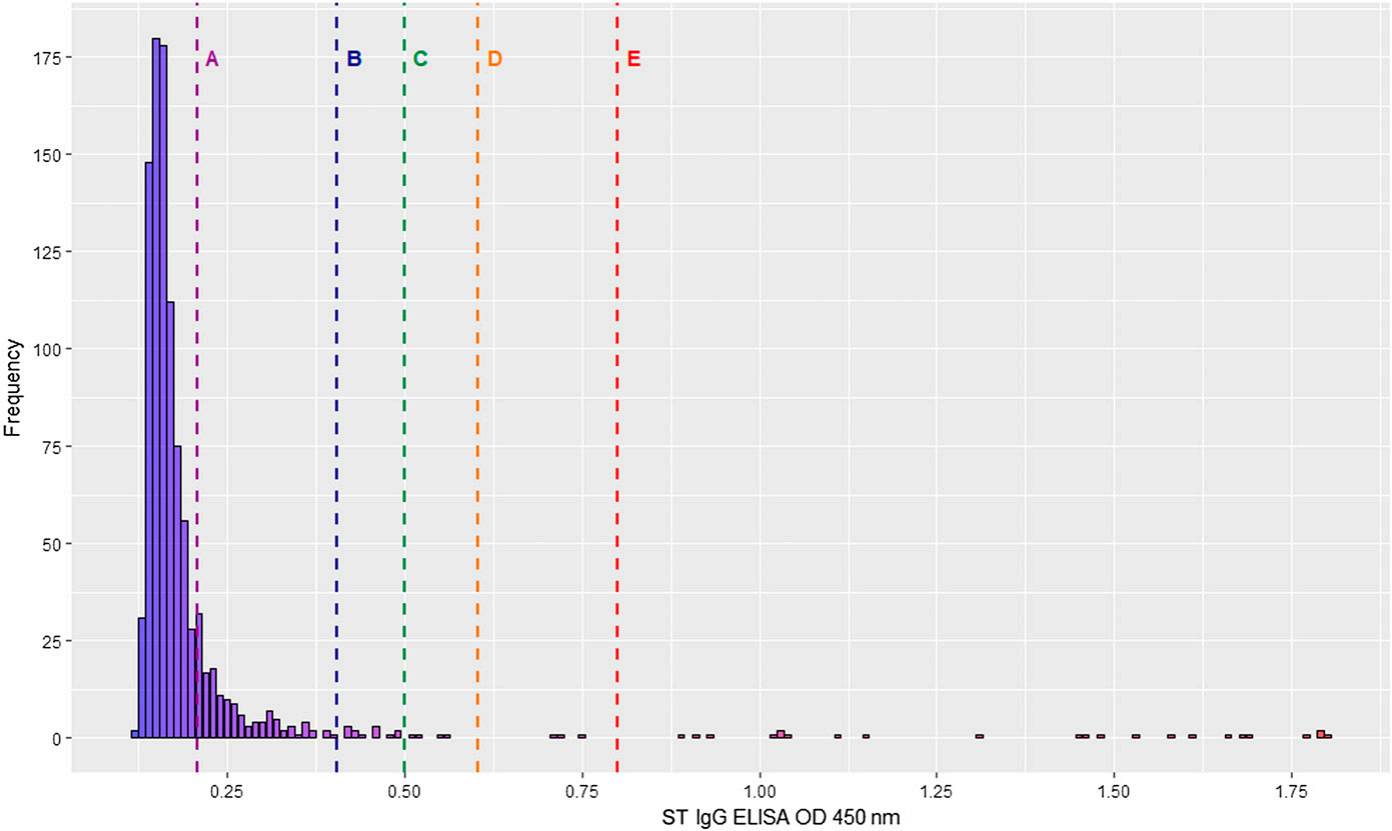
Histogram of ST IgG ELISA OD distribution. Vertical lines represent different OD cutoffs for determining seropositivity: Line A, mean OD (0.21); Line B, mean OD plus 1 SD (0.20); Line C, OD 0.50; Line D, mean OD plus 2 SD; Line E, mean OD plus 3 SD. OD = optical density; ST = Scrub typhus. This figure appears in color at www.ajtmh.org.

**Figure 2.**
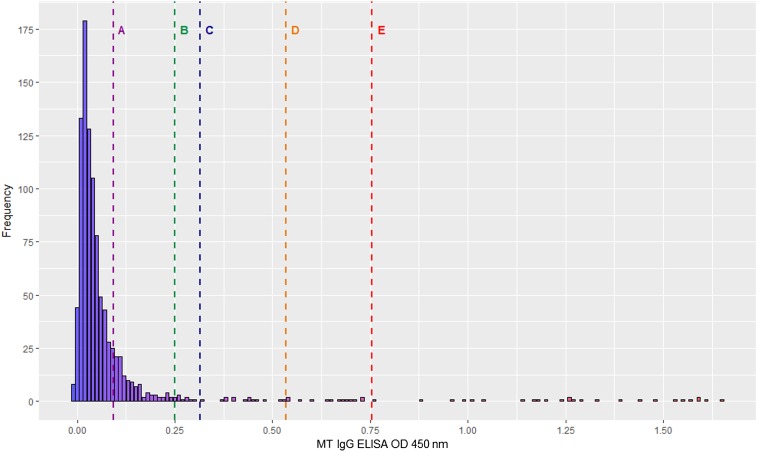
Histogram of MT IgG ELISA OD distribution. Vertical lines represent different OD cutoffs for determining seropositivity: Line A, mean OD (0.09); Line B, OD 0.25; Line C, mean OD plus 1 SD (0.22); Line D, mean OD plus 2 SD; Line E, mean OD plus 3 SD. MT = murine typhus; OD = optical density. This figure appears in color at www.ajtmh.org.

Ethical approval was obtained from the AHC Institutional Review Board (AHC-IRB, 091-17), the Oxford Tropical Research Ethics Committee (OxTREC, 5123-16), and the Cambodian National Ethics Committee for Health Research (NECHR, 083).

A total of 1,268 serum samples were collected. Of these, 1,007 samples were submitted for IgG testing after deduplication. Of the 1,007 tested samples, 14 were excluded because of metadata errors. All 993 samples were tested for the presence of ST and MT IgG. Because of ELISA kit unavailability, only 837 (84.3%) samples could be tested for DENV IgG: all samples from 0- to 5-year-olds and the first 45 samples per year of life for older children.

Of the 993 analyzable samples, there were 46–72 per patient year of life, with 496 samples (50.0%) from male children. The overall seroprevalence of ST, MT, and DENV was estimated to be 4.2%, 5.3%, and 50.7%, respectively (Table 1). Considering a range of potential OD cutoffs (Figures 1 and 2), seroprevalence estimates for ST and MT were 2.3–17.0% and 2.6–17.8%, respectively.

**Table 1:** Scrub typhus, murine typhus, and dengue virus seroprevalence (proportion seropositive) by age group

Age group (years)	Scrub typhus			Murine typhus			Dengue virus		
	No. sera positive/no. sera tested	Percent seropositive	95% CI (%)	No. sera positive/no. sera tested	Percent seropositive	95% CI (%)	No. sera positive/no. sera tested	Percent seropositive	95% CI (%)
0–3	6/270	2.2	0.8–4.8	12/270	4.4	2.3–7.6	74/270	27.4	22.2–33.1
4–7	9/225	4.0	1.8–7.5	9/225	4.0	1.8–7.5	84/202	41.6	34.7–48.7
8–11	15/240	6.3	3.5–10.1	9/240	3.8	1.7–7.0	114/179	63.7	56.2–70.7
12–15	12/258	4.7	2.4–8.0	23/258	8.9	5.7–13.1	152/186	81.7	75.4–87.0
Total	42/993	4.2	3.1–5.7	53/993	5.3	4.0–6.9	424/837	50.7	47.2–54.1

Age-associated patterns of seropositivity varied by pathogen (Table 1). For ST, seropositivity rose from 2.2% in 0- to 3-year-olds to 4.0% in 4- to 7-year-olds, before peaking at 6.3% in 8- to 11-year-olds and dropping to 4.7% in 12- to 15-year-olds. Conversely, MT seropositivity remained at approximately 4% in children of age groups 0–3, 4–7, and 8–11 years, before rising to 8.9% in 12- to 15-year-olds. Dengue virus showed a steady seropositivity increase by age group, rising from 27.4% in 0- to 3-year-olds to 81.7% in 12- to 15-year-olds.

Analyzing by year of life revealed distinct age-related peaks in seropositivity for the different pathogens (Supplemental Table 1). Scrub typhus seropositivity ranged from 1.4% to 6.7% in 0- to 8-year-olds, then peaked at 12.5% in 9-year-olds, before dropping again to 1.4–7.8% in 10- to 15-year-olds. Murine typhus seropositivity was 9.7% in 0- to 11-month-olds, before dropping to 0.0% in 1-year-olds, and then rising to 10.7% in 6-year-olds, and 11.1% and 10.3% in 13- and 14-year-olds, respectively. Dengue virus seropositivity was 66.7% in 0- to 11-month-olds before dropping to 5.7% in 1-year-olds and then steadily increasing by age to reach 87.0% seropositivity in 15-year-olds.

Little is known about pediatric ST and MT seroprevalence in Southeast Asia, although the seroprevalence seen here in Cambodian children (4.2% and 5.3%, respectively) is similar to that of adults in Thailand (4.2% for both pathogens).^9^ The high DENV seroprevalence seen here (50.7%) is similar to that of children in neighboring Thailand (55.4–62.5%, 59%, and 71%)^10–12^ and Vietnam (54.9% and 65.7%),^13,14^ and higher than that of children in Laos (20–29.6%).^15^

A similar seroprevalence of ∼4–5% for both ST and MT contrasts with their relative contribution to pediatric admissions with febrile illness of 7.8% and 2.2%, respectively.^3^ This suggests that although both rickettsial infections may have similar prevalence, ST may lead to a higher health-care burden. The reasons for this require further exploration but may be related to greater clinical severity or duration of acute ST infection compared with MT, leading to increased health-care–seeking behavior and/or hospitalization rates.

Scrub typhus seropositivity peaked in the 8- to 11-year age group and was highest in 9-year-olds, suggesting these may be more common ages for contracting initial ST infection. Conversely, MT seropositivity peaked in the 12- to 15-year age group, particularly in 13- to 14-year-olds, suggesting initial infection with MT may commonly occur in this older age group.

The steady increase in DENV seroprevalence by age indicates constant levels of DENV exposure, with long-lasting immunity and IgG levels that are stable over time. However, these levels should be interpreted cautiously: they may be overestimates due to cross-reactivity with other anti-flavivirus immunoglobulins, such as Japanese encephalitis, a common childhood pathogen in Cambodia for which children are now routinely immunized.^3,16^

Scrub typhus and MT seroprevalence did not increase linearly with age, suggesting ST and MT immunity is short-lasting. Studies of ST-specific IgG kinetics following acute infection found that IgG levels were short lived, and waned to baseline after 2–3 years.^6,17^ This is consistent with the findings of this study, which demonstrated discrete peaks in ST- and MT-specific IgG levels, possibly corresponding to ages at which acute infection occurs, but then no overall increase in IgG levels with age. These findings have important implications for vaccine development.

High levels of seropositivity in < 1-year-olds for MT and DENV suggest that vertical transfer of maternal MT- and DENV-specific IgG occurs. This is evident to a high degree for DENV, with ∼2/3 of < 1-year-olds seropositive, and to a lesser extent for MT, with ∼1/10 of < 1-year-olds seropositive. This is corroborated by studies showing efficient transplacental transfer of DENV-specific antibodies^18,19^ and animal models showing newborn acquisition of DENV-specific IgG via breast milk.^20^ The same phenomenon was not observed for ST, with low levels of seropositivity in < 1-year-olds, suggesting either an absence of maternal ST-specific IgG or a lack of vertical transfer of these antibodies. More research is needed to investigate maternal transmission of anti-rickettsial antibodies.

The high overall seroprevalence of ST, MT, and DENV indicates that these are common and significant pathogens in Cambodian children, with approximately one in 20–25 children infected by ST or MT, and half of children infected by DENV. The results of this study are of both local and regional importance in improving care of children presenting with febrile illness in Southeast Asia. A greater understanding of the seroprevalence of these pathogens and age of initial infection could impact diagnostic and treatment algorithms and guide prospective vaccine development. These findings suggest that in Cambodian children presenting with undifferentiated febrile illness, dengue should generally be considered high in the list of differential diagnoses and empirical anti-rickettsial antimicrobial therapy may be more indicated in 8- to 15-year-olds than in children aged 7 years and less.

## Supplementary Files

Supplemental table
