# Supplementary material for: Seroprevalence of Dengue Virus and Rickettsial Infections in Cambodian Children
Source: Am J Trop Med Hyg. 2019 Jan 21;100(3):635–8. doi: 10.4269/ajtmh.18-0865 (PMC6402902; doi:10.4269/ajtmh.18-0865)
Supplement: Supplementary file 1 [file tpmd180865.SD1.pdf]

The following are supplemental materials and will be published online only

Supplementary Table 1.
Scrub typhus, murine typhus and dengue virus seroprevalence (proportion seropositive) by year of life.

Age (years)	Scrub typhus			Murine typhus			Dengue virus		
	No. sera positive/no. sera tested	% seropositive	95% CI (%)	No. sera positive/no. sera tested	% seropositive	95% CI (%)	No. sera positive/no. sera tested	% seropositive	95% CI (%)
0	1/72	1.4	0.0-7.5	7/72	9.7	4.0-19.0	48/72	66.7	54.6-77.3
1	1/70	1.4	0.0-7.7	0/70	0.0	0.0-5.1	4/70	5.7	1.6-14.0
2	3/69	4.3	0.9-12.2	1/69	1.4	0.0-7.8	5/69	7.2	2.4-16.1
3	1/59	1.7	0.0-9.1	4/59	6.8	1.9-16.5	17/59	28.8	17.8-42.1
4	4/60	6.7	1.8-16.2	2/60	3.3	0.4-11.5	14/60	23.3	13.4-36.0
5	1/55	1.8	0.0-9.7	1/55	1.8	0.0-9.7	22/55	40.0	27.0-54.1
6	1/56	1.8	0.0-9.6	6/56	10.7	4.0-21.9	21/42	50.0	34.2-65.8
7	3/54	5.6	1.2-15.4	0/54	0.0	0.0-6.6	27/45	60.0	44.3-74.3
8	2/57	3.5	0.4-12.1	3/57	5.3	1.1-14.6	25/46	54.3	39.0-69.1
9	7/56	12.5	5.2-24.1	2/56	3.6	0.4-12.3	30/43	69.8	53.9-82.8
10	5/64	7.8	2.6-17.3	1/64	1.6	0.0-8.4	29/46	63.0	47.5-76.8
11	1/63	1.6	0.0-8.5	3/63	4.8	1.0-13.3	30/44	68.2	52.4-81.4
12	1/72	1.4	0.0-7.5	5/72	6.9	2.3-15.5	38/48	79.2	65.0-89.5
13	5/72	6.9	2.3-15.5	8/72	11.1	4.9-20.7	34/45	75.6	60.5-87.1
14	3/68	4.4	0.9-12.4	7/68	10.3	4.2-20.1	40/47	85.1	71.7-93.8
15	3/46	6.5	1.4-17.9	3/46	6.5	1.4-17.9	40/46	87.0	73.7-95.1
Total	42/993	4.2	3.1-5.7	53/993	5.3	4.0-6.9	424/837	50.7	47.2-54.1

CI = confidence interval.
